# Retraction Note to: Inhibition of TGF-β repairs spinal cord injury by attenuating EphrinB2 expressing through inducing miR-484 from fibroblast

**DOI:** 10.1038/s41420-022-01159-2

**Published:** 2022-08-18

**Authors:** Dayu Pan, Fuhan Yang, Shibo Zhu, Yongjin Li, Guangzhi Ning, Shiqing Feng

**Affiliations:** 1 Department of Orthopedics, Tianjin Medical University General Hospital, Heping District, Tianjin, 300052 PR China; 2 International Science and Technology Cooperation Base of Spinal Cord Injury, Tianjin Key Laboratory of Spine and Spinal Cord Injury, Department of Orthopedics, Tianjin Medical University General Hospital, Tianjin, China; 3 Department of Urology, Shanghai Tenth People’s Hospital, Tongji University, Shanghai, 200072 China

**Keywords:** Diseases of the nervous system, Spinal cord injury

Retraction to: *Cell Death Discovery* 10.1038/s41420-021-00705-8, published online 28 Oct 2021

The Editors-in-Chief have retracted this article at the authors’ request. After publication, the authors became aware of significant issues with the data presented here. Specifically:

- The authors have found that the described effect of 1D11 TGF-b neutralizing antibody on fibrotic scar formation after spinal cord injury appears to be time-dependent and further experiments do not support the results and conclusions presented in this paper.
- The authors have used the staining of EphrinB2, a ligand, to measure the expression of Eph Receptor B2 (EphB2, a receptor). This is incorrect and readers should disregard the data and conclusions based on this part of the study.
- As per article title, the project investigated miR-484, but it was referred to as miR-488 throughout the text by mistake.

The authors have lost confidence in the results and conclusions presented in this article.

